# Fatty acid-binding protein 1 increases steer fat deposition by facilitating the synthesis and secretion of triacylglycerol in liver

**DOI:** 10.1371/journal.pone.0214144

**Published:** 2019-04-22

**Authors:** Yujuan Wang, Keqiong Tang, Wei Zhang, Wenli Guo, Yaning Wang, Linsen Zan, Wucai Yang

**Affiliations:** 1 College of Animal Science and Technology, Northwest A & F University, Yangling, Shaanxi, China; 2 College of Veterinary Medicine, Northwest A&F University, Yangling, Shaanxi, China; 3 National Beef Cattle Improvement Centre, Yangling, Shaanxi, China; University of Illinois, UNITED STATES

## Abstract

Castration is an important means of improving the beef quality via increasing fat deposition. However, little is known about the molecular mechanism underlying the fat deposition after castration. Here, the intramuscular fat (IMF) content of the steer group was shown to be much higher than the bull group. To understand transcriptional changes in the genes involved in fat deposition following castration, differential expression patterns of mRNAs in liver tissue were investigated in steers and bulls using RNA sequencing. In total, we obtained 58,282,367–54,918,002 uniquely mapped reads, which covered 90.13% of the currently annotated transcripts; 5,864 novel transcripts and optimized 9,088 known genes were determined. These results indicated that castration could change the expression patterns of mRNAs in liver tissue, and 282 differentially expressed genes (DEGs) were detected between steers and bulls. KEGG pathway analysis showed that the DEGs were mostly enriched in PPAR signaling pathway, steroid biosynthesis, steroid hormone biosynthesis, and biosynthesis of fatty acids. Furthermore, eight DEGs were corroborated via quantitative real-time PCR and we found that *FABP1* gene knockdown in bovine hepatocytes prominently reduced intracellular triacylglycerol (TAG) synthesis and very low density lipoprotein (VLDL) secretion in culture medium. In summary, these results indicate that *FABP1* may promote fat deposition by promoting the production and secretion of TAG and VLDL in steer liver.

## Introduction

Castration is an important means of improving beef quality via increasing fat deposition, and thus raising prices at market compared with the carcasses from bulls [1–4]. Therefore, castration has been proposed as a method in the beef industry improve beef quality, and the number of castrated male livestock is now increasing due to their high market value [5]. However, studies of the mechanisms and regulation of fat deposition after castration are limited. The major sites of lipogenesis are adipose tissue and the liver [6, 7], and recent studies have indicated that liver tissue participates in various metabolic processes and plays a crucial role in regulating lipometabolism [8]. Therefore, sequencing of the liver transcriptome between bulls and steers can effectively analyze its functional complexity.

With the advancement of high-throughput sequencing technology, liver transcriptome sequencing results have been analyzed and many potential candidate genes affecting fat deposition in pig, chicken and cattle have been discovered [9]. Wang *et al*. (2017) analyzed the liver transcriptome of Simmental and Jinnan bulls and detected 124 DEGs, which participate in the regulation of lipid metabolism [9]. Li *et al*. (2015) compared the liver transcriptome profiling between juvenile hens and laying hens and 960 DEGs were obtained; KEGG pathway analysis showed that the DEGs were most enriched in lipid biosynthesis [10]. Xing *et al*. (2015) identified 92 DEGs in liver tissue between pigs with higher and lower backfat thickness, related to lipid metabolism, regulation, and transport [11]. However, a review of researches in the past decades showed an insufficiency of liver transcriptome research between bulls and steers.

FABP1 is liver-specific fatty acid-binding protein (FABP) that plays important roles in intracellular lipid metabolism in the liver [12]. *In vitro* cell models and *in vivo* mouse models have indicated that FABP1 plays an important role in regulating hepatic lipid metabolism. *FABP1* overexpression significantly increased hepatocyte fatty acid uptake [13], *de novo* lipogenesis [14], and VLDL secretion [15, 16], whereas knockdown of *FABP1* remarkably blocked lipid accumulation in hepatocytes [17]. *FABP1* knockout mice had significantly decreased liver weight and hepatic TAG accumulation [14], and which indicated that pharmacological agents that attenuate *FABP1* expression or function may suppress TAG accumulation in the liver [8, 14, 16].

In this study, the expression profiles of liver lipid metabolism-related genes were investigated between bulls and steers using RNA-Seq technology. Bioinformatics tools were used to analyze the major DEGs and pathways that might contribute to fat deposition after castration. In addition, small interfering RNA (siRNA) was used to elucidate the functional roles of DEGs in hepatic lipid metabolism. The purpose of this study was to reveal the mechanism of lipid metabolism related genes in *bovine* liver. These findings will be a valuable resource to improve the comprehensive of castration mechanism in altering fat deposition.

## Materials and methods

### Ethics statement

This study was conducted in strict accordance with the Regulations for the Administration of Affairs Concerning Experimental Animals (Ministry of Science and Technology, China, revised 2004). The protocol was approved by the Committee on the Ethics of Animal Experiments of the Laboratory Animals of Northwest A&F University. All surgery was performed under sodium pentobarbital anesthesia, and all efforts were made to minimize suffering.

### Sample preparation and RNA extraction

Six Qinchuan bull born within a 30-day period were randomly selected to be unrelated for at least three generations, and three of these six bull calves were castrated at 6 months of age. The cattle were raised and maintained under the same condition at the National Beef Cattle Improvement Centre (Yangling, China). The sternomandibularis muscle tissue of each animal was sampled, and quickly dissected intramuscular fat (IMF) tissue. IMF content was analyzed as described earlier [18]. Liver tissue was immediately collected from 24 months old steers and bulls. All tissue samples were instantly put into liquid nitrogen and then stored at -80°C for°C the next experiment. Total RNA was extracted from collected liver tissues using Trizol reagent (TaKaRa, Dalian, China). Aggregate RNA was extracted from the same group and pooled before constructing an index library for Illumina sequencing.

### Sequencing data analysis

Low quality reads, those containing adapters and poly-N, were eliminated from raw reads acquired from the Illumina sequencing in order to get clean reads. We calculated Q30, GC content, and sequence duplication level of the clean data. Then, we mapped the clean reads to the reference genome of Bos taurus (version UMD 3.1.1) using Tophat2 software [19]. Further analysis and annotation based on the reference genome was only performed if there was an exact match or one mismatch.

### Quantitative analysis and differential expression analysis of gene expression

The differential abundance of gene expression was defined by fragments per kilo base of transcript per million fragments mapped reads (FPKM) using cufflinks. Identification of differentially expressed genes (DEGs) between bull liver (BL) and steer liver (SL) was accomplished in the DESeq R package(1.10.1) based on a negative binomial distribution [20]. In order to control the error discovery rate, the *P* values were rectified by the Benjamini and Hochberg approach [21]. Genes with fold changes ≥ 2 or FDR value ≤ 0.05 were identified as significantly differentially expressed genes [22]. Real-time PCR primers for amplification of mRNAs were designed by Primer Premier 5.0 and synthesized by ShengGong (Songon Biotech; S1 Table). Quantitative real-time PCR was performed using SYBR Premix EX Taq II (Takara) and (Tiangen) in a 7500 Real-Time PCR system (Applied Biosystems Inc., Foster City, CA). The relative expression results were obtained using the 2^−ΔΔCt^ method.

### Enrichment analysis of functions and signaling pathways in the differentially expressed genes

Enrichment analysis of KEGG pathway categories and GO biological process terms of the DEGs were analyzed using the web-based tools in DAVID [23]. The ensembl gene IDs of DEGs in bovine were uploaded to database for enrichment analysis of the significantly overrepresented KEGG pathway categories and GO biological process terms. Only the GO terms and KEGG pathways with *P*≤0.05 were taken into account as significantly enriched among the DEGs.

### Cell culture

Liver tissue samples were obtained from nine fetal calves and samples from three calves were mixed under aseptic conditions. Isolation of hepatocytes was conducted using the collagenase IV perfusion method according to the protocol described by Li *et al*. (2014) and Shi *et al*. (2015) [24,25]. Hepatocytes were cultivated in complete growth medium including F-12 base medium containing 10% fetal bovine serum, 1% pnicillin and streptomycin. Hepatocytes were maintained at 37°C and 5% CO_2_. Cells were trypsinized with 0.25% trypsin and then passaged into six-well cell culture plates when grew to 80% confluence.

### siRNA transfection

Hepatocytes were transfected with siRNAs when the cell-density reached 70%. FABP1 siRNAs and control siRNAs synthesized by ShengGong (Songon Biotech) were transfected at a final concentration of 50 nM using Lipofectamine RNAiMAX (Invitrogen) according to the manufacturer’s instructions. Cells were collected for analysis 48 h after transfection.

### Total RNA extraction and real-time PCR

Total RNA was extracted from cultured hepatocytes using Trizol reagent (TaKaRa, Dalian, China) according to the protocol issued by the manufacturer. For mRNA expression analysis, first-strand cDNA was synthesized using a reverse transcription kit (TaKaRa, Dalian, China) and GAPDH was used as an endogenous control gene. Real-time PCR primers (S1 Table) for amplification of mRNA were designed by NCBI (National Center for Biotechnology Information, USA) and synthesized by TSINGKE Biological technology (TSINGKE Biological technology, Xi'an, China). Quantitative real-time PCR was performed using SYBR Premix EX Taq II (Takara) in 7500 Real-Time PCR system (Applied Biosystems Inc., Foster City, CA). The relative expression levels of mRNAs were obtained via the 2^−ΔΔCt^ method [26].

### TAG content assay

The intracellular TAG content was detected using tissue triglyceride assay kit (Applygen Technologies, Beijing, China). A BCA assay kit was used to determine the hepatocyte protein content (Sangon Biotech). Perform all experiments base on the protocols recommended by the manufacturers. The content of triglycerides in total cellular protein concentrations were normalized and the results were denoted as micrograms per milligram of protein.

### VLDL content assay

The culture medium was collected after 48 h of treatment and stored at -20°C for further analysis. The concentration of VLDL were detected using the VLDL assay kit (Shanghai Enzyme-linked Biotech, Shanghai, China) based on the manufacturer protocol.

### Statistical analysis

All data are expressed as the mean ± SEM. Statistical differences between groups were assessed by Student’s t-test via SPSS Statistics 17 software (SPSS Inc., Chicago, IL). Statistical significance was declared at P < 0.05 and P < 0.01.

## Results

### IMF content and transcriptome map of liver tissues in *steer* and *bull*

The IMF content of steer liver group (SL) was 4.08%, which was significantly higher than 3.19% in the bull liver group (BL) (Published in [27]). We established six cDNA libraries from the SL (n:3) and BL (n:3) groups. Solexa sequencing respectively provided 66,210,326 and 60,933,394 reads from the BL and SL libraries. In total, 59,682,779 reads were matched to the bovine genome in the BL library and 55,801,528 reads were from the SL library (Table 1). After assembly, 5,864 novel transcripts and optimized 9,088 known genes were obtained from these two groups.

**Table 1:** Summary of transcriptome sequencing data.

**Sample name**	**Total reads**	**Total mapped**	**Uniquely mapped**	**Non-splice reads**	**Splice reads**
**BL**	66,210,326	59,682,779 (90.14%)	58,282,367 (88.03%)	33,154,965 (50.08%)	25,127,402 (37.95%)
**SL**	60,933,394	55,801,528 (91.58%)	54,918,002 (90.13%)	30,866,108 (50.66%)	24,051,894 (39.47%)

Note: BL, bull liver. SL, steer liver.

### Differentially expressed genes between BL and SL groups

**Fig 1:**
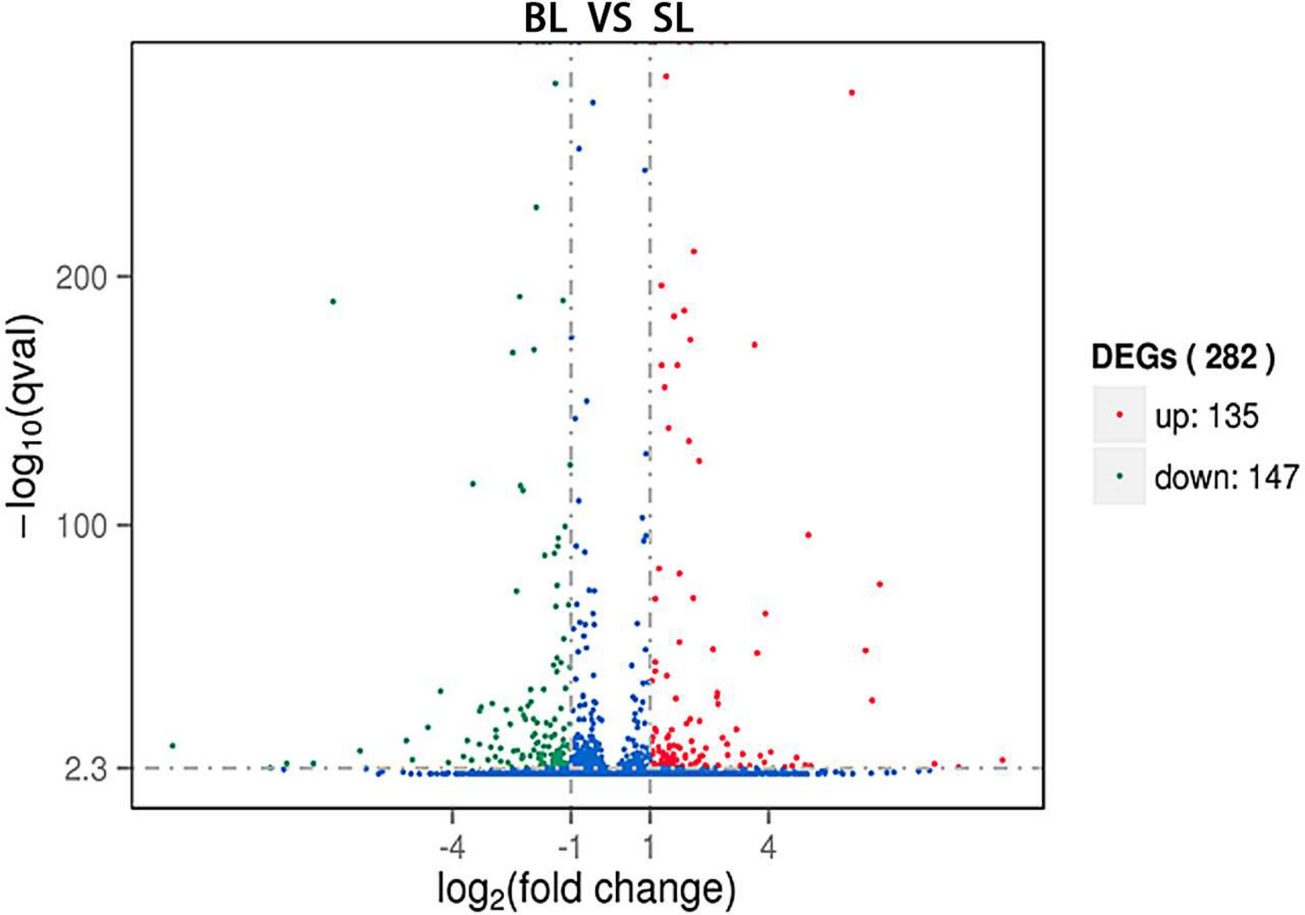
The differential expression of bovine mRNAs between BL and SL tissues. Note: Each point in the figure represents a mRNA. Red points represent up-expressed mRNAs; blue points represent equally-expressed mRNAs; green points represent down-expressed mRNAs. BL = bull liver; SL = steer liver; n = 3 replicates per group.

We compared the liver tissues from bulls and steers and found that the mRNA expression levels were different. We detected 282 DEGs (Fig 1), of which 135 genes were up-regulated and 147 genes were down-regulated; there were 17 novel genes (with 15 up-regulated and 2 down-regulated in the BL group) (S2 Table) between the BL and SL groups.

**Fig 2:**
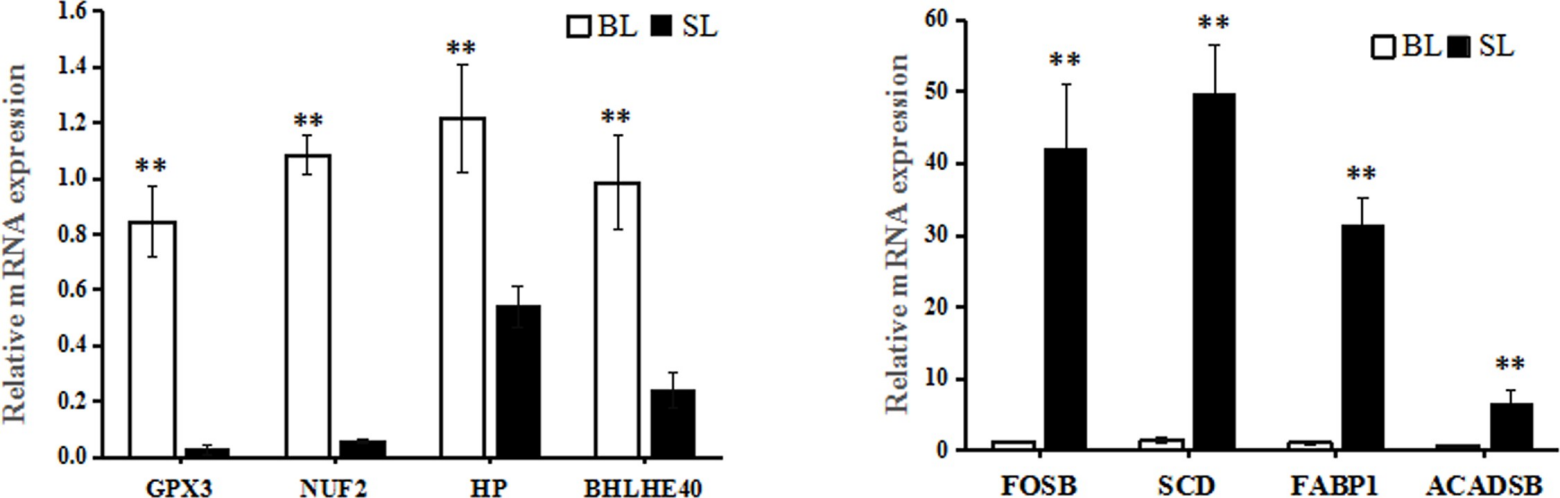
Different expression levels of eight mRNAs in BL and SL. The values are presented as means ± S.E.M. Statistically significant differences are indicated: **P < 0.01. BL = bull liver; SL = steer liver.

To confirm changes in transcript levels, eight genes were validated in BL and SL groups. The expression levels of *GPX3*, *NUF2*, *HP*, and *BHLHE40* were significantly higher in bulls than in steers, and the *FOSB*, *SCD*, *FABP1*, and *ACADSB* expression levels were higher in steers (Fig 2); the qPCR results and Solexa sequencing were consistent.

### Enrichment analysis of functions and signaling pathways in the differentially expressed genes

The GO and KEGG pathway enrichment analysis of DEGs were performed in order to acquire the biological relationships of DEGs in liver between bulls and steers. There were 25 GO biological process terms significantly enriched (*P* < 0.05), which included fatty acid metabolic process (GO: 0006631), lipid metabolic process (GO: 0006629), and monocarboxylic acid metabolic process (GO: 0032787) (Table 2).

**Table 2:** Summary of the GO analysis of 25 differently expressed genes.

GO ID	GO term	No. of DEGs	P-value
**GO:0044710**	single-organism metabolic process	64	0.0001
**GO:0055114**	oxidation-reduction process	36	0.0003
**GO:0016491**	oxidoreductase activity	37	0.0003
**GO:0004866**	endopeptidase inhibitor activity	10	0.0003
**GO:0061135**	endopeptidase regulator activity	10	0.0003
**GO:0002526**	acute inflammatory response	4	0.0005
**GO:0006953**	acute-phase response	4	0.0005
**GO:0048037**	cofactor binding	18	0.0010
**GO:0042612**	MHC class I protein complex	4	0.0010
**GO:0005615**	extracellular space	11	0.0021
**GO:0030414**	peptidase inhibitor activity	10	0.0021
**GO:0061134**	peptidase regulator activity	10	0.0021
**GO:0016616**	oxidoreductase activity, acting on the CH-OH group of donors, NAD or NADP as acceptor	11	0.0031
**GO:0016614**	oxidoreductase activity, acting on CH-OH group of donors	11	0.0055
**GO:0009611**	response to wounding	9	0.0072
**GO:0006631**	fatty acid metabolic process	8	0.0082
**GO:0004857**	enzyme inhibitor activity	11	0.0097
**GO:0006954**	inflammatory response	4	0.0100
**GO:0050660**	flavin adenine dinucleotide binding	8	0.0101
**GO:0006629**	lipid metabolic process	23	0.0101
**GO:0016705**	oxidoreductase activity, acting on paired donors, with incorporation or reduction of molecular oxygen	12	0.0109
**GO:0050662**	coenzyme binding	13	0.0235
**GO:0032787**	monocarboxylic acid metabolic process	8	0.0246
**GO:0020037**	heme binding	9	0.0327
**GO:0005506**	iron ion binding	10	0.0393

**Fig 3:**
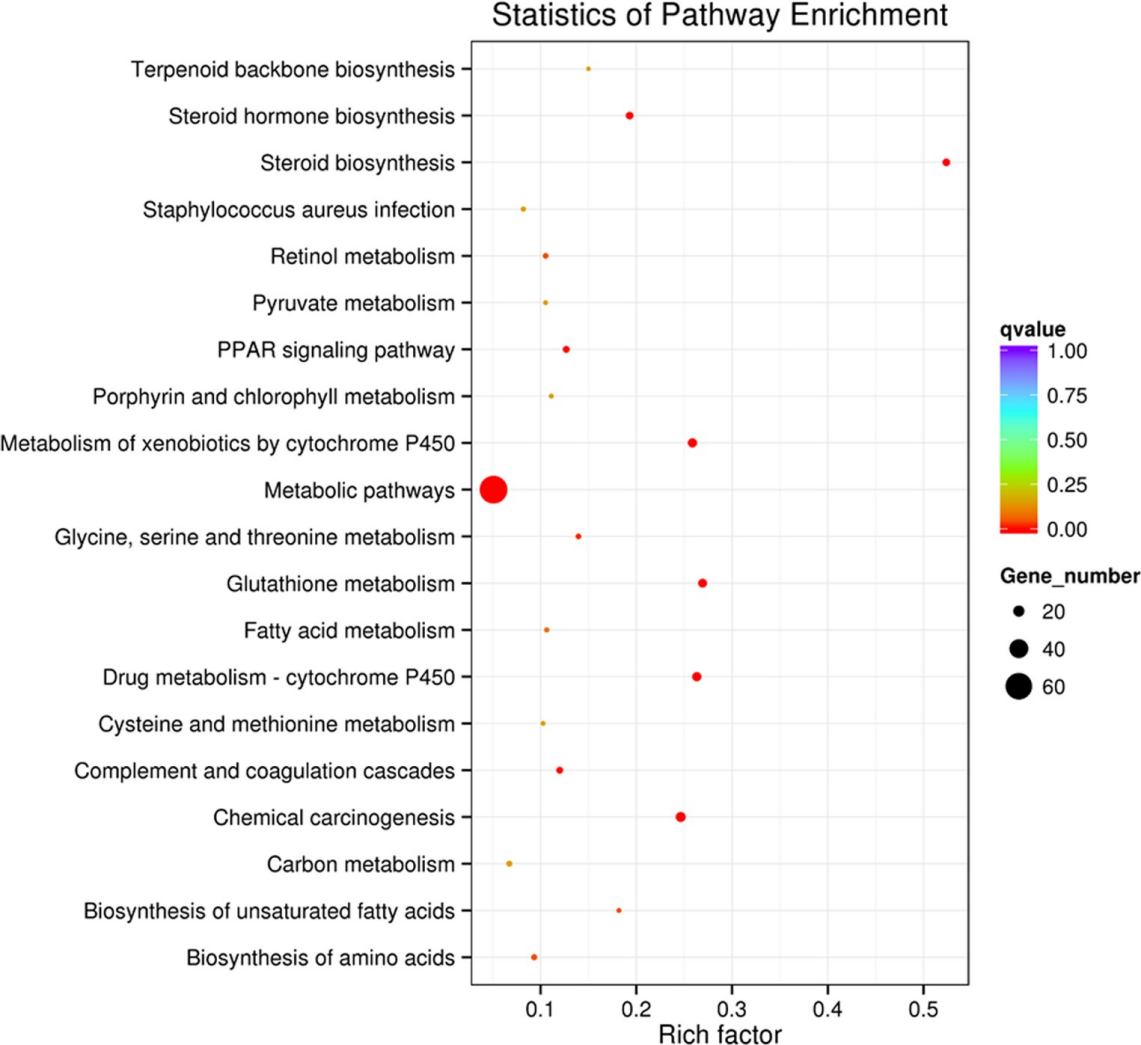
KEGG pathway analysis of the differentially expressed genes between BL and SL. BL = bull liver; SL = steer liver.

Thirty KEGG pathways were significantly enriched (*P*<0.05), including PPAR signaling pathway, steroid biosynthesis, steroid hormone biosynthesis, and biosynthesis of fatty acids (Fig 3).

### *FABP1* gene silencing decrease the content of TAG and VLDL

**Fig 4:**
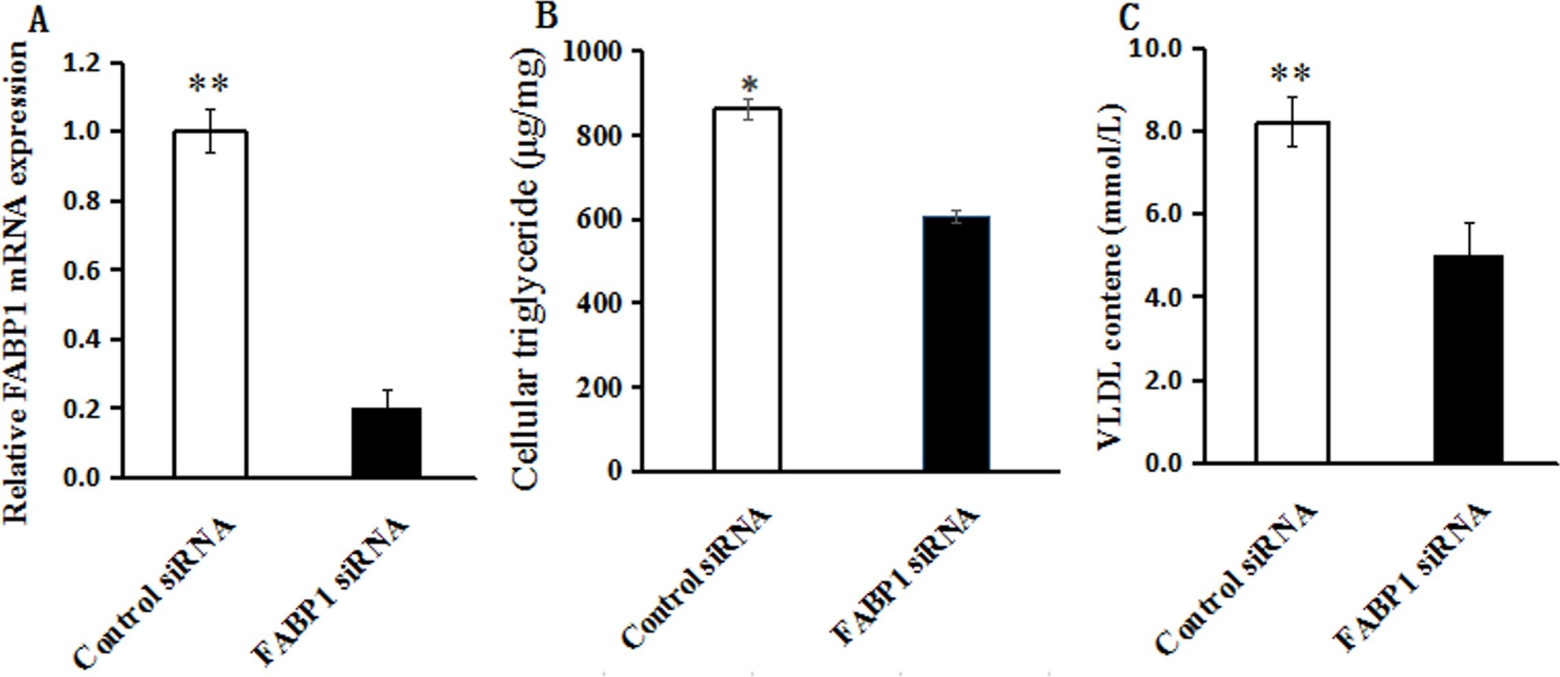
(A) Relative mRNA expression levels of *FABP1* in bovine hepatocytes. Cells were collected after 48 hours of transfection with control or *FABP1* siRNA. (B) The content of cellular TAG changed by FABP1 silencing in *bovine* hepatocytes.(C) The content of extracellular VLDL changed by FABP1 silencing in bovine hepatocytes. The values are presented as means ± S.E.M. Statistically significant differences are indicated: *P < 0.05, **P < 0.01. The experiments were done in three biological replicates and two technical replicates.

The *FABP1* gene was silenced with specific siRNA to identify the role of FABP1 in lipid metabolism in hepatocytes. The hepatocytes were transfected with 50 nM siRNA and negative control, and the knockout efficiency was 80% (Fig 4A). Compared with the control group, *FABP1* gene knockdown significantly decreased cellular TAG accumulation and inhibited VLDL secretion in culture medium (Fig 4B and 4C).

## Discussion

There have been many studies to assess the differences in meat characteristics between bulls and steers and it is now widely believed that castration can increase the fat content in the back and intramuscular of the carcass [1, 3]. Castration of male animals reduces the concentration of circulating androgen. Previous studies in mice, pig and beef cattle have shown a causal relationship between the low levels of testosterone and excess fat deposition accumulation [28,29]. With the advancement of high-throughput sequencing technology, an association of mRNAs with fat synthesis induced by testosterone deficiency via castration has been reported in skeletal and adipose tissue in pig and cattle [28,29]. The liver plays a key role in lipid metabolism, hence, analyses of liver transcriptome in pig, chicken, and bovine with distinct IMF deposition have been performed to explore potential candidate genes affecting fat deposition [9–11]. However, the variation of the mRNA expression profiles caused by castration in liver tissue and its effects on fat deposition are still unrevealed in bovine.

In the current study, we employed RNA-seq to explore whole transcriptome expression differences in the liver tissue between bulls and steers. We identified 282 DEGs, of which 135 genes were up-regulated and 147 genes were down-regulated. It should be noticed that many DEGs were related to lipid metabolism. For example, *APOA12*, *APOA2*, *FADS2*, *SC4MOL*, and *SCD* had higher expression in SL than BL. Previous studies have revealed that *APOA1* and *APOA2* genes were key regulatory factors of high density lipoprotein metabolism, which is significantly associated with obesity and body weight in humans [30]; *APOA1* and *APOA2* have been considered as candidate genes for back fat thickness in pigs [30]. The *FADS2* gene regulates unsaturation of fatty acids and influences fat-related traits and has been found to be significantly associated with beef quality traits [31]. The *SC4MOL* gene is involved in cholesterol biosynthesis [32], and the *SCD* gene is significantly associated with fatty acid composition in Japanese Black cattle [33]. Lipid metabolism is a complex process that is controlled by multiple pathways and genes. In this study, *KEGG* analysis showed that the DEGs were mostly enriched in the *PPAR* signaling pathway, steroid biosynthesis, steroid hormone biosynthesis, and biosynthesis of fatty acids, which played an important role in lipid metabolism [10, 27, 34]. The results indicated that these DEGs could be considered as key candidate genes regulating fatty acid deposition in steer. However, clarifying their roles in fat deposition still require further research.

Fatty acid-binding proteins (FABPs) are members of the superfamily of lipid-binding proteins [35], which have been shown to be central to lipid-mediated processes and related metabolic [36]. A-FABP is a adipocyte-specific FABP that plays important roles in intracellular trafficking of fatty acids [36, 37], and has been demonstrated to be one of the major metabolic indicators of animals' ability to deposit IMF [37]. FABP1 is a liver-specific fatty acid-binding protein [38]. *In vitro* cell models and in vivo mouse models have indicated that FABP1 plays an important role in regulating fatty acid uptake [13], *de novo* lipogenesis [14], TAG accumulation [14, 17] and VLDL secretion [15, 16]. In this study, the expression level of FABP1 gene in *steers* was significantly higher than that of bulls and *FABP1* gene silencing in bovine hepatocytes significantly reduced TAG accumulation and decreased secretion of VLDL. Previous studies in ruminants, especially in cattle, revealed that hepatic TAG synthesis is a consequence of multifarious processes of lipid metabolism, including fatty acids *de novo* synthesis and secretion of TAG via VLDL [39–41]. These results indicated that *FABP1* may promote the accumulation of fat deposition via the production and secretion of TAG and VLDL in bovine liver [14, 39–41].

## Supporting information

S1 Table Primers for quantitative real-time PCR. (DOCX)

S2 Table Differentially expressed mRNAs between the bull (BL) and steer liver (SL) tissue. (DOCX)
