# Supplementary material for: Fatty acid-binding protein 1 increases steer fat deposition by facilitating the synthesis and secretion of triacylglycerol in liver
Source: PLoS One. 2019 Apr 22;14(4):e0214144. doi: 10.1371/journal.pone.0214144 (PMC6476475; doi:10.1371/journal.pone.0214144)
Supplement: S1 Table — (DOCX) [file pone.0214144.s001.docx]

| **Table S1. Primers for quantitative real-time PCR.** | | | |
| --- | --- | --- | --- |
| **Genes** | **Primer sequence** | **T_ann_(℃)** |  |
| **GPX3** | F:TAGCCACCCTCAAGTATGTTCG | 61℃ |  |
|  | R:CGAGGTAGGAGGACAGGAGT |  |  |
| **NUF2** | F:AAAGGAGAGCCTGAACTTGGAG | 61℃ |  |
|  | R:TCTGAACTGTGCTGTGGCAA |  |  |
| **HP** | F:CCCTGAATGTGAGGCAGTGTG | 61℃ |  |
|  | R:TTAGCTGTGGTGAGGAGCCA |  |  |
| **BHLHE40** | F:GGACAGCAAGGAGACCTACAA | 61℃ |  |
|  | R:GTTTGAGATGTTCGGGGAGGA |  |  |
| **FOSB** | F:CGAAGTGTAGGAACCGGCGA | 61℃ |  |
|  | R:CACAAACTCCAGACGTTCCTTC |  |  |
| **SCD** | F:TGGTGAATAGTGCTGCCCAT | 61℃ |  |
|  | R:GGTGGTAGTTGTGGAAGCCC |  |  |
| **FABP1** | F:AGACCCAGGAGAACTATGAGG | 61℃ |  |
|  | R:GCCAGCGGTGATGATGAACTT |  |  |
| **ACADSB** | F:AGGCACGTCTTCGCGGTATC | 61℃ |  |
|  | R:ATTGCTTGTCGCTTTGAGCTG |  |  |
| **β-actin** | F:CACCAACTGGGACGACAT | 61℃ |  |
|  | R:ATACAGGGACAGCACAGC |  |  |
