# Supplementary material for: Fatty acid-binding protein 1 increases steer fat deposition by facilitating the synthesis and secretion of triacylglycerol in liver
Source: PLoS One. 2019 Apr 22;14(4):e0214144. doi: 10.1371/journal.pone.0214144 (PMC6476475; doi:10.1371/journal.pone.0214144)
Supplement: S2 Table — (DOCX) [file pone.0214144.s002.docx]

**Table S2. Differentially expressed mRNAs between the bull (BL) and steer liver (SL) tissue.**

|  | | | | | |
| --- | --- | --- | --- | --- | --- |
| **Gene_id** | **readcount_BL** | **readcount_SL** | **log2.Fold _Change** | **pvalue** | **geneName** |
|  |  |  |  |  |  |
| ENSBTAG00000000259 | 1810.386597 | 447.0280476 | 2.0179 | 5.62E-178 | CHIA |
| ENSBTAG00000000342 | 125.638785 | 61.2128243 | 1.0374 | 1.21E-05 | PLOD3 |
| ENSBTAG00000000396 | 158.0082501 | 48.75932062 | 1.6962 | 1.32E-13 | PIM1 |
| ENSBTAG00000000575 | 1425.42839 | 360.8676508 | 1.9819 | 4.20E-137 | TNC |
| ENSBTAG00000001448 | 104.5036662 | 14.68458741 | 2.8312 | 3.49E-17 | INMT |
| ENSBTAG00000001511 | 64.33481691 | 25.15039832 | 1.355 | 7.77E-05 | BCL6 |
| ENSBTAG00000001595 | 501.261978 | 230.0855795 | 1.1234 | 6.00E-21 | MT1E |
| ENSBTAG00000001638 | 49465.2303 | 22637.42731 | 1.1277 | 0 | FGA |
| ENSBTAG00000001927 | 42.06818116 | 3.042386894 | 3.7895 | 9.99E-10 | ATP6V1C2 |
| ENSBTAG00000001936 | 1188.416015 | 542.6806915 | 1.1309 | 6.13E-48 | PCK1 |
| ENSBTAG00000002069 | 346.6886899 | 3.082952052 | 6.8132 | 2.34E-79 | BOLA |
| ENSBTAG00000002191 | 47.96823347 | 2.880126259 | 4.0579 | 2.41E-11 | RRP12 |
| ENSBTAG00000002214 | 1093.77271 | 499.1948415 | 1.1316 | 2.61E-44 | TAT |
| ENSBTAG00000002255 | 1136.245005 | 240.4296949 | 2.2406 | 4.12E-129 | BHMT |
| ENSBTAG00000002258 | 296.8211245 | 142.221446 | 1.0615 | 6.52E-12 | APOA1 |
| ENSBTAG00000002333 | 435.4319424 | 13.58932813 | 5.0019 | 3.06E-99 | HOP |
| ENSBTAG00000002340 | 428.8448977 | 210.8171292 | 1.0245 | 1.31E-15 | STEAP4 |
| ENSBTAG00000002473 | 86.23775081 | 26.97583046 | 1.6767 | 5.90E-08 | ANGPTL4 |
| ENSBTAG00000003492 | 331.4536233 | 21.90518564 | 3.9195 | 1.77E-67 | - |
| ENSBTAG00000003851 | 200.3593105 | 71.80033069 | 1.4805 | 6.52E-14 | CCNL1 |
| ENSBTAG00000004118 | 93.59261054 | 44.70280476 | 1.066 | 0.00010829 | ALAS1 |
| ENSBTAG00000004269 | 447.7573941 | 213.2104735 | 1.0704 | 1.90E-17 | SGK1 |
| ENSBTAG00000004278 | 85.95487159 | 35.41338344 | 1.2793 | 1.35E-05 | APMAP |
| ENSBTAG00000004305 | 229.091757 | 35.49451376 | 2.6903 | 7.11E-34 | RGS16 |
| ENSBTAG00000004680 | 238.1843033 | 59.10343606 | 2.0108 | 7.96E-25 | SLC13A5 |
| ENSBTAG00000005146 | 54.31281026 | 6.977207276 | 2.9606 | 4.92E-10 | - |
| ENSBTAG00000005586 | 2319.44796 | 836.0073533 | 1.4722 | 1.99E-142 | GATM |
| ENSBTAG00000005754 | 131.8621279 | 43.93206675 | 1.5857 | 1.35E-10 | PPM1K |
| ENSBTAG00000006157 | 49.14016166 | 11.11485345 | 2.1444 | 1.05E-06 | HS6ST1 |
| ENSBTAG00000006354 | 36796.4036 | 4862.870081 | 2.9197 | 0 | HP |
| ENSBTAG00000006745 | 42792.67526 | 20574.5673 | 1.0565 | 0 | FGG |
| ENSBTAG00000006864 | 1150.712256 | 559.2718414 | 1.0409 | 2.67E-40 | - |
| ENSBTAG00000006921 | 285.3038991 | 97.51864124 | 1.5487 | 1.80E-20 | ABCA6 |
| ENSBTAG00000006934 | 55.44432714 | 11.84502631 | 2.2268 | 1.05E-07 | CYP11A1 |
| ENSBTAG00000007041 | 704.2480241 | 165.4247167 | 2.0899 | 9.11E-74 | - |
| ENSBTAG00000007043 | 4919.02717 | 1228.110176 | 2.0019 | 0 | - |
| ENSBTAG00000007077 | 196.3181788 | 41.09250565 | 2.2562 | 6.00E-24 | ABHD1 |
| ENSBTAG00000007214 | 106.2413528 | 41.98493913 | 1.3394 | 4.93E-07 | ELL2 |
| ENSBTAG00000007247 | 16.40699477 | 0 | 4.4119 | 6.31E-05 | NUF2 |
| ENSBTAG00000007850 | 7440.935828 | 2237.330757 | 1.7337 | 0 | ITIH4 |
| ENSBTAG00000008039 | 69.02252971 | 23.00044492 | 1.5854 | 3.39E-06 | ACMSD |
| ENSBTAG00000008101 | 113.8386804 | 42.47172104 | 1.4224 | 4.48E-08 | PCSK5 |
| ENSBTAG00000008339 | 151.0575035 | 56.50726591 | 1.4186 | 3.21E-10 | DHX8 |
| ENSBTAG00000008441 | 70.11363527 | 14.64402225 | 2.2594 | 1.58E-09 | SOCS3 |
| ENSBTAG00000008462 | 27.8029862 | 1.865997295 | 3.8972 | 5.25E-07 | FAM43A |
| ENSBTAG00000008571 | 83.28772466 | 22.47309786 | 1.8899 | 5.79E-09 | CUX2 |
| ENSBTAG00000009493 | 94.11795766 | 25.31265896 | 1.8946 | 5.62E-10 | BCL3 |
| ENSBTAG00000009583 | 19.68031146 | 0 | 4.9223 | 7.33E-06 | SLC22A2 |
| ENSBTAG00000009735 | 2663.914027 | 873.8952114 | 1.608 | 1.83E-187 | A1BG |
| ENSBTAG00000009798 | 47.84699951 | 13.58932813 | 1.816 | 1.81E-05 | DCDC2 |
| ENSBTAG00000009863 | 206.3401854 | 31.31630243 | 2.72 | 4.86E-31 | BHLHE40 |
| ENSBTAG00000010515 | 109.2317903 | 52.57244553 | 1.055 | 3.43E-05 | FBXW5 |
| ENSBTAG00000011374 | 67.28484307 | 24.05513904 | 1.4839 | 1.35E-05 | S1PR3 |
| ENSBTAG00000011525 | 83.40895861 | 10.79033218 | 2.9505 | 1.40E-14 | SMCT1 |
| ENSBTAG00000011952 | 223.6362292 | 2.555604991 | 6.4513 | 1.37E-52 | SULT1E1 |
| ENSBTAG00000012046 | 151.5828507 | 73.30124156 | 1.0482 | 1.22E-06 | JUNB |
| ENSBTAG00000012078 | 66.03209224 | 24.70418158 | 1.4184 | 3.23E-05 | TMEM176A |
| ENSBTAG00000012189 | 29.13655967 | 4.421602286 | 2.7202 | 1.34E-05 | TMEM86B |
| ENSBTAG00000012210 | 4055.962669 | 1657.330119 | 1.2912 | 6.26E-200 | C5 |
| ENSBTAG00000012263 | 22.91321683 | 2.352779198 | 3.2837 | 2.14E-05 | ASAP3 |
| ENSBTAG00000012508 | 220.1608559 | 93.58382085 | 1.2342 | 1.43E-11 | OSGIN1 |
| ENSBTAG00000012854 | 71.97255586 | 28.07108974 | 1.3584 | 2.82E-05 | GSDMB |
| ENSBTAG00000013391 | 106.1201189 | 35.16999249 | 1.5933 | 7.36E-09 | ANKH |
| ENSBTAG00000013578 | 18.67002853 | 0 | 4.5983 | 1.63E-05 | CHI3L2 |
| ENSBTAG00000013819 | 96.50222537 | 46.00088983 | 1.0689 | 8.13E-05 | URAD |
| ENSBTAG00000015557 | 706.3090013 | 262.9433579 | 1.4255 | 1.92E-42 | - |
| ENSBTAG00000015711 | 233.6178245 | 61.57791073 | 1.9237 | 5.26E-23 | BTG2 |
| ENSBTAG00000016388 | 113.2325107 | 39.91611605 | 1.5042 | 1.14E-08 | SLCO4A1 |
| ENSBTAG00000016766 | 91.81451258 | 17.28075756 | 2.4096 | 6.25E-13 | TMEM176B |
| ENSBTAG00000016864 | 1870.276169 | 798.1600603 | 1.2285 | 1.03E-85 | LBP |
| ENSBTAG00000017814 | 77.79178553 | 27.05696078 | 1.5236 | 1.76E-06 | LGI4 |
| ENSBTAG00000018137 | 2157.034876 | 591.5617076 | 1.8664 | 9.32E-190 | A2M |
| ENSBTAG00000018473 | 99.00772704 | 41.45759207 | 1.2559 | 4.27E-06 | MARCO |
| ENSBTAG00000019460 | 121.8401212 | 40.40289795 | 1.5925 | 5.88E-10 | MOXD1 |
| ENSBTAG00000019603 | 690.5485876 | 206.8823088 | 1.7389 | 4.70E-56 | LDHB |
| ENSBTAG00000019616 | 432.4010936 | 137.5970179 | 1.6519 | 3.36E-33 | APCS |
| ENSBTAG00000019700 | 1036.71193 | 308.9848129 | 1.7464 | 9.11E-84 | PC |
| ENSBTAG00000019798 | 184.5584855 | 65.99951302 | 1.4836 | 5.71E-13 | PIGR |
| ENSBTAG00000019929 | 113.4749786 | 46.77162785 | 1.2787 | 5.79E-07 | ITGAV |
| ENSBTAG00000020244 | 108.7468545 | 52.73470616 | 1.0441 | 4.21E-05 | EFNA1 |
| ENSBTAG00000020558 | 252.2474417 | 119.707783 | 1.0753 | 1.50E-10 | APOC2 |
| ENSBTAG00000020979 | 66.43620541 | 25.92113634 | 1.3578 | 5.76E-05 | NGFR |
| ENSBTAG00000021050 | 135.5395577 | 1.379215392 | 6.6187 | 2.28E-32 | ECEL1 |
| ENSBTAG00000021180 | 142.8540062 | 42.3094604 | 1.7555 | 5.03E-13 | TBATA |
| ENSBTAG00000021407 | 18.18509272 | 0 | 8.8083 | 7.38E-05 | CASP16 |
| ENSBTAG00000021452 | 152.3506657 | 58.73834963 | 1.375 | 7.51E-10 | TRANK1 |
| ENSBTAG00000021964 | 45.01820731 | 14.60345709 | 1.6242 | 0.0001319 | CDH17 |
| ENSBTAG00000022120 | 65130.19246 | 32111.2984 | 1.0202 | 0 | FGB |
| ENSBTAG00000022394 | 928.5308343 | 74.39650084 | 3.6416 | 6.60E-176 | SAA1 |
| ENSBTAG00000022395 | 2746.716816 | 467.837974 | 2.5536 | 0 | - |
| ENSBTAG00000022396 | 41.82571326 | 13.02141591 | 1.6835 | 0.00015211 | SAA3 |
| ENSBTAG00000022520 | 156.7554993 | 62.51090938 | 1.3263 | 1.37E-09 | BRCA1 |
| ENSBTAG00000023941 | 95.04741795 | 39.95668121 | 1.2502 | 7.21E-06 | CPN1 |
| ENSBTAG00000024700 | 141.8841345 | 34.277559 | 2.0494 | 8.59E-16 | - |
| ENSBTAG00000025462 | 186.7811079 | 74.27480537 | 1.3304 | 3.34E-11 | GADD45B |
| ENSBTAG00000026275 | 50.67579172 | 4.989514506 | 3.3443 | 1.85E-10 | CCL24 |
| ENSBTAG00000026917 | 4997.950472 | 1881.939402 | 1.4091 | 3.35E-284 | - |
| ENSBTAG00000027516 | 46.02849024 | 11.31767925 | 2.0239 | 5.54E-06 | APLNR |
| ENSBTAG00000030246 | 96.78510459 | 43.12076358 | 1.1664 | 2.01E-05 | ENTPD8 |
| ENSBTAG00000030913 | 81.18633617 | 37.07655495 | 1.1307 | 0.00014455 | MX1 |
| ENSBTAG00000034633 | 266.6742819 | 114.9210943 | 1.2144 | 2.27E-13 | ERO1LB |
| ENSBTAG00000034985 | 128.1442867 | 52.73470616 | 1.2809 | 1.04E-07 | PHLDA1 |
| ENSBTAG00000037743 | 23.84267712 | 0 | 8.1991 | 2.71E-06 | C1R |
| ENSBTAG00000037778 | 178.0926747 | 58.576089 | 1.6042 | 5.02E-14 | CXCL3 |
| ENSBTAG00000038067 | 2198.335242 | 679.385276 | 1.6941 | 9.11E-168 | MT1A |
| ENSBTAG00000038430 | 113.1112767 | 12.45350369 | 3.1831 | 1.31E-20 | -//- |
| ENSBTAG00000038706 | 63.3649453 | 23.08157523 | 1.4569 | 3.16E-05 | MT1E |
| ENSBTAG00000039688 | 69.7095221 | 13.95441455 | 2.3206 | 9.18E-10 | FAM101A |
| ENSBTAG00000040409 | 2909.776481 | 1125.034108 | 1.3709 | 7.15E-159 | - |
| ENSBTAG00000040413 | 450.0204279 | 202.0144897 | 1.1555 | 7.44E-20 | CES3 |
| ENSBTAG00000043553 | 1206.843576 | 17.56471367 | 6.1024 | 1.30E-277 | GPX3 |
| ENSBTAG00000043567 | 381.8061245 | 63.24108223 | 2.5939 | 4.26E-53 | -//- |
| ENSBTAG00000043570 | 1849.504752 | 845.3779049 | 1.1295 | 1.80E-73 | -//- |
| ENSBTAG00000043582 | 261.582456 | 20.03918834 | 3.7064 | 1.39E-51 | -//- |
| ENSBTAG00000044106 | 100.664591 | 23.64948745 | 2.0897 | 6.53E-12 | SPIDR |
| ENSBTAG00000045822 | 136.1861388 | 65.83725238 | 1.0486 | 4.21E-06 | -//- |
| ENSBTAG00000045904 | 76.94314787 | 28.92295807 | 1.4116 | 7.84E-06 | PIM3 |
| ENSBTAG00000046540 | 9507.287708 | 2303.614226 | 2.0451 | 0 | SERPINA3-1 |
| ENSBTAG00000047040 | 2048.449667 | 474.5312251 | 2.11 | 1.13E-213 | SERPINA3-6 |
| ENSBTAG00000048094 | 3390.954034 | 1384.286037 | 1.2925 | 1.03E-167 | - |
| Novel00122 | 46.14972419 | 13.79215392 | 1.7425 | 4.44E-05 | -//- |
| Novel00205 | 68.73965049 | 26.04283181 | 1.4003 | 2.71E-05 | -//- |
| Novel00223 | 67.28484307 | 26.81356982 | 1.3273 | 7.13E-05 | -//- |
| Novel00322 | 17.6597456 | 0 | 5.0656 | 1.99E-05 | -//- |
| Novel00355 | 53.86828577 | 18.74110327 | 1.5232 | 6.99E-05 | -//- |
| Novel00456 | 34.30920827 | 6.530990532 | 2.3932 | 1.19E-05 | -//- |
| Novel00585 | 272.7763908 | 98.89785663 | 1.4637 | 4.40E-18 | -//- |
| Novel00705 | 29.25779362 | 5.43573125 | 2.4283 | 4.49E-05 | -//- |
| Novel00747 | 77.58972894 | 26.2456576 | 1.5638 | 1.10E-06 | -//- |
| Novel00892 | 239.2349976 | 36.83316399 | 2.6994 | 1.99E-35 | -//- |
| Novel00941 | 21.41799809 | 1.865997295 | 3.5208 | 2.28E-05 | -//- |
| Novel00993 | 39.19897764 | 0 | 9.9164 | 6.21E-08 | -//- |
| Novel00995 | 134.4080409 | 62.26751843 | 1.1101 | 1.51E-06 | -//- |
| Novel01003 | 33.9050951 | 1.298085075 | 4.707 | 5.31E-09 | -//- |
| Novel01005 | 265.6235877 | 98.28937925 | 1.4343 | 3.97E-17 | -//- |
| ENSBTAG00000000087 | 29.45985021 | 95.65264394 | -1.6991 | 1.90E-10 | HSD17B12 |
| ENSBTAG00000000103 | 91.93574653 | 212.2774749 | -1.2073 | 7.23E-14 | MRPL23 |
| ENSBTAG00000000140 | 436.7655158 | 992.6699957 | -1.1845 | 2.26E-57 | EPHX1 |
| ENSBTAG00000000163 | 25.58036376 | 57.72422067 | -1.1741 | 0.00013127 | DDIT4 |
| ENSBTAG00000000507 | 4.243188302 | 24.0957042 | -2.5056 | 5.41E-05 | NR4A1 |
| ENSBTAG00000000522 | 1329.613157 | 4801.21104 | -1.8524 | 0 | AHSG |
| ENSBTAG00000000836 | 87.77338087 | 233.2496619 | -1.41 | 1.90E-18 | KRT8 |
| ENSBTAG00000001265 | 192.6407489 | 896.6522653 | -2.2186 | 2.96E-117 | HRG |
| ENSBTAG00000001488 | 119.3750309 | 254.4246746 | -1.0917 | 2.72E-14 | OTC |
| ENSBTAG00000001658 | 118.6880385 | 316.9761492 | -1.4172 | 1.16E-24 | AKR1D1 |
| ENSBTAG00000001660 | 29.17697099 | 93.01590863 | -1.6726 | 5.08E-10 | SPTY2D1 |
| ENSBTAG00000001842 | 49.86756537 | 120.3568255 | -1.2711 | 4.96E-09 | GSTM3 |
| ENSBTAG00000001992 | 41.82571326 | 192.8873291 | -2.2053 | 1.99E-26 | CYP51A1 |
| ENSBTAG00000002006 | 21.74128863 | 75.45119497 | -1.7951 | 4.54E-09 | THBS1 |
| ENSBTAG00000002705 | 0 | 42.5122862 | -6.3385 | 7.66E-12 | REC8 |
| ENSBTAG00000003068 | 53.10047075 | 592.9003579 | -3.481 | 6.55E-120 | SC4MOL |
| ENSBTAG00000003234 | 5.859640988 | 35.49451376 | -2.5987 | 5.89E-07 | HSD17B2 |
| ENSBTAG00000003329 | 4.162365667 | 35.73790471 | -3.102 | 4.90E-08 | FST |
| ENSBTAG00000003548 | 6.748689965 | 67.0947723 | -3.3135 | 1.55E-14 | GSTP1 |
| ENSBTAG00000003746 | 447.6765715 | 1141.057346 | -1.3498 | 7.37E-79 | SCP2 |
| ENSBTAG00000003758 | 41.90653589 | 158.2446836 | -1.9169 | 8.51E-19 | TKT |
| ENSBTAG00000003846 | 230.1424512 | 468.0002346 | -1.024 | 7.32E-23 | RPL37A |
| ENSBTAG00000003948 | 36.89553256 | 144.1685736 | -1.9662 | 9.11E-18 | FDPS |
| ENSBTAG00000004038 | 79.97399665 | 246.7172945 | -1.6253 | 2.99E-23 | ABAT |
| ENSBTAG00000004075 | 37.74417023 | 134.1489794 | -1.8295 | 2.49E-15 | IDI1 |
| ENSBTAG00000004288 | 20.4885378 | 72.00315649 | -1.8132 | 8.17E-09 | GSTA4 |
| ENSBTAG00000004303 | 57.464893 | 208.3832196 | -1.8585 | 2.21E-23 | SLC27A2 |
| ENSBTAG00000004322 | 18.34673799 | 60.96943335 | -1.7326 | 2.59E-07 | FOS |
| ENSBTAG00000004688 | 42.55311697 | 204.9757463 | -2.2681 | 7.58E-29 | DHCR24 |
| ENSBTAG00000005069 | 58.75805515 | 150.3750429 | -1.3557 | 7.69E-12 | TM7SF2 |
| ENSBTAG00000005479 | 111.9797598 | 257.1019751 | -1.1991 | 2.49E-16 | LIPC |
| ENSBTAG00000005498 | 15.23506657 | 147.0081347 | -3.2704 | 1.12E-29 | SQLE |
| ENSBTAG00000005574 | 852.5171468 | 1888.186437 | -1.1472 | 1.10E-102 | CLU |
| ENSBTAG00000005596 | 14.58848549 | 108.9985811 | -2.9014 | 2.40E-20 | IGFBP2 |
| ENSBTAG00000006353 | 26.59064669 | 90.70369459 | -1.7702 | 1.88E-10 | TXNL4B |
| ENSBTAG00000006546 | 103.0084474 | 536.393092 | -2.3805 | 1.24E-76 | GSTA2 |
| ENSBTAG00000006599 | 109.7167261 | 237.8740899 | -1.1164 | 7.34E-14 | INHBE |
| ENSBTAG00000006795 | 41.17913218 | 90.46030364 | -1.1354 | 3.00E-06 | GCSH |
| ENSBTAG00000007115 | 33.09686875 | 69.52868181 | -1.0709 | 8.78E-05 | GSR |
| ENSBTAG00000007375 | 12.32545173 | 42.22833009 | -1.7766 | 1.32E-05 | MIF |
| ENSBTAG00000007390 | 42.27023775 | 114.8805291 | -1.4424 | 4.17E-10 | VAT1 |
| ENSBTAG00000007829 | 14.66930813 | 75.24836917 | -2.3589 | 5.07E-12 | CSAD |
| ENSBTAG00000007840 | 27.8029862 | 172.6453149 | -2.6345 | 1.34E-28 | HMGCR |
| ENSBTAG00000007846 | 700.6514169 | 1459.656101 | -1.0589 | 4.20E-71 | ITIH3 |
| ENSBTAG00000008091 | 33.78386114 | 68.87963928 | -1.0277 | 0.00015301 | SELENBP1 |
| ENSBTAG00000008103 | 410.8618615 | 1574.212109 | -1.9379 | 5.05E-174 | ALDH1A1 |
| ENSBTAG00000008109 | 58.03065144 | 182.0564317 | -1.6495 | 6.86E-18 | TMEM97 |
| ENSBTAG00000008127 | 29.05573704 | 80.35957916 | -1.4676 | 1.23E-07 | RBP5 |
| ENSBTAG00000008160 | 1.414396101 | 24.25796483 | -4.1002 | 6.88E-07 | MBOAT2 |
| ENSBTAG00000008182 | 1.656864003 | 59.46852248 | -5.1656 | 5.12E-16 | FOSB |
| ENSBTAG00000008203 | 14.58848549 | 41.49815723 | -1.5082 | 0.00010779 | SEPW1 |
| ENSBTAG00000008253 | 43.36134331 | 98.28937925 | -1.1806 | 5.44E-07 | EXPH5 |
| ENSBTAG00000008353 | 118.4859819 | 382.4483151 | -1.6905 | 6.27E-37 | CDKN1A |
| ENSBTAG00000008587 | 41.82571326 | 185.5856005 | -2.1496 | 8.73E-25 | GSTT3 |
| ENSBTAG00000009212 | 1453.231376 | 3348.49158 | -1.2042 | 7.87E-194 | APOA2 |
| ENSBTAG00000009231 | 34.47085354 | 126.3199038 | -1.8736 | 6.34E-15 | NSDHL |
| ENSBTAG00000009287 | 39.27980028 | 111.026839 | -1.499 | 2.83E-10 | EBP |
| ENSBTAG00000009570 | 16.7302853 | 53.62713965 | -1.6805 | 2.19E-06 | C19orf80 |
| ENSBTAG00000009615 | 66.71908463 | 164.4105877 | -1.3011 | 3.64E-12 | ANXA2 |
| ENSBTAG00000009844 | 14.18437232 | 43.93206675 | -1.631 | 2.66E-05 | CYR61 |
| ENSBTAG00000010069 | 9.941184021 | 68.43342253 | -2.7832 | 7.01E-13 | EGR1 |
| ENSBTAG00000010508 | 44.41203756 | 110.5806223 | -1.3161 | 9.09E-09 | BLVRB |
| ENSBTAG00000010564 | 17.57892296 | 56.46670075 | -1.6836 | 1.15E-06 | MGC139109 |
| ENSBTAG00000010801 | 55.68679504 | 236.9410913 | -2.0891 | 3.34E-30 | CMBL |
| ENSBTAG00000010841 | 90.1980599 | 259.4141891 | -1.5241 | 1.81E-22 | FMO5 |
| ENSBTAG00000010913 | 5.25347123 | 35.94073051 | -2.7743 | 2.05E-07 | SRXN1 |
| ENSBTAG00000010991 | 1384.61296 | 4517.295495 | -1.706 | 0 | TTR |
| ENSBTAG00000011021 | 591.5004492 | 2179.038624 | -1.8812 | 1.82E-231 | CES1 |
| ENSBTAG00000011027 | 152.3102544 | 345.0066738 | -1.1796 | 6.50E-21 | PTGR1 |
| ENSBTAG00000011839 | 21.09470756 | 167.4935398 | -2.9892 | 3.40E-31 | HMGCS1 |
| ENSBTAG00000012012 | 226.1417308 | 611.2763747 | -1.4346 | 9.30E-47 | CYB5A |
| ENSBTAG00000012432 | 53.34293865 | 134.4735007 | -1.334 | 1.57E-10 | FDFT1 |
| ENSBTAG00000013303 | 5.980874939 | 44.98676087 | -2.9111 | 2.73E-09 | ACSS2 |
| ENSBTAG00000013960 | 65.30468853 | 262.9433579 | -2.0095 | 7.04E-32 | PSAT1 |
| ENSBTAG00000013981 | 30.47013314 | 75.24836917 | -1.3043 | 2.47E-06 | SPTSSA |
| ENSBTAG00000015214 | 24.16596766 | 140.4771442 | -2.5393 | 9.48E-23 | CA3 |
| ENSBTAG00000015505 | 6.384988111 | 826.474541 | -7.0161 | 1.96E-193 | FADS2 |
| ENSBTAG00000015509 | 29.50026153 | 62.79486549 | -1.0899 | 0.00015921 | NAMPT |
| ENSBTAG00000015571 | 20.36730385 | 71.55693974 | -1.8128 | 9.13E-09 | GCLC |
| ENSBTAG00000015654 | 182.9420328 | 885.5779771 | -2.2752 | 3.58E-119 | PON1 |
| ENSBTAG00000016275 | 23.72144317 | 59.38739217 | -1.324 | 2.34E-05 | AMDHD1 |
| ENSBTAG00000016465 | 13.13367808 | 60.80717272 | -2.211 | 2.21E-09 | DHCR7 |
| ENSBTAG00000016472 | 10.66858773 | 36.42751241 | -1.7717 | 5.35E-05 | GSTM2 |
| ENSBTAG00000016542 | 190.1756585 | 452.4232137 | -1.2503 | 3.79E-29 | LAMB3 |
| ENSBTAG00000016771 | 48.45316927 | 100.9666797 | -1.0592 | 2.76E-06 | PLK2 |
| ENSBTAG00000017121 | 21849.22726 | 63369.95774 | -1.5362 | 0 | ALB |
| ENSBTAG00000017181 | 35.68319305 | 76.42475877 | -1.0988 | 2.78E-05 | MACROD1 |
| ENSBTAG00000017531 | 54.27239894 | 209.5190441 | -1.9488 | 7.63E-25 | FETUB |
| ENSBTAG00000017602 | 28.24751069 | 75.73515108 | -1.4228 | 5.04E-07 | TMEM45B |
| ENSBTAG00000017765 | 24.85296005 | 95.53094847 | -1.9426 | 4.04E-12 | GSTM1 |
| ENSBTAG00000017863 | 21.78169995 | 60.44208629 | -1.4724 | 4.29E-06 | SRGN |
| ENSBTAG00000018041 | 2.747969567 | 31.39743274 | -3.5142 | 7.60E-08 | ACADSB |
| ENSBTAG00000018054 | 212.2402377 | 1177.322598 | -2.4717 | 8.87E-173 | FABP1 |
| ENSBTAG00000018365 | 556.0193128 | 1400.755491 | -1.333 | 1.01E-94 | CYP2E1 |
| ENSBTAG00000018382 | 22.38786971 | 52.61301068 | -1.2327 | 0.00015547 | SMS |
| ENSBTAG00000018509 | 43.52298858 | 96.30168648 | -1.1458 | 1.22E-06 | ETNK2 |
| ENSBTAG00000018556 | 487.3200736 | 986.5852219 | -1.0176 | 6.17E-46 | MGC137211 |
| ENSBTAG00000018872 | 34.91537802 | 87.53961222 | -1.3261 | 2.74E-07 | F12 |
| ENSBTAG00000018936 | 43.96751307 | 113.0145318 | -1.362 | 2.64E-09 | LSS |
| ENSBTAG00000019246 | 314.2384022 | 751.9563447 | -1.2588 | 9.15E-48 | SC5DL |
| ENSBTAG00000019782 | 55.04021397 | 113.2579228 | -1.0411 | 9.50E-07 | TPI1 |
| ENSBTAG00000020116 | 41.62365667 | 99.54689917 | -1.258 | 1.30E-07 | JSP.1 |
| ENSBTAG00000020406 | 8.082263432 | 65.9183827 | -3.0278 | 2.31E-13 | GPC3 |
| ENSBTAG00000020789 | 2.869203518 | 37.88785812 | -3.723 | 1.67E-09 | STS |
| ENSBTAG00000021102 | 53.50458392 | 131.9584609 | -1.3023 | 4.63E-10 | GALM |
| ENSBTAG00000021386 | 119.4962648 | 242.2956922 | -1.0198 | 1.66E-12 | ANGPTL3 |
| ENSBTAG00000021408 | 277.262047 | 706.8884535 | -1.3502 | 1.48E-49 | FMO1 |
| ENSBTAG00000021516 | 294.4772681 | 1446.51299 | -2.2964 | 1.91E-195 | GSTA1 |
| ENSBTAG00000021934 | 35.23866856 | 81.00862169 | -1.2009 | 4.12E-06 | RETSAT |
| ENSBTAG00000022246 | 72.41708035 | 204.0833128 | -1.4948 | 1.40E-17 | C29H11orf86 |
| ENSBTAG00000022590 | 11.43640276 | 54.27618219 | -2.2467 | 1.17E-08 | BOLA |
| ENSBTAG00000023384 | 40.73460769 | 131.4311138 | -1.69 | 1.01E-13 | - |
| ENSBTAG00000023549 | 462.9924607 | 1240.401419 | -1.4217 | 6.83E-92 | CYP2C19 |
| ENSBTAG00000025280 | 45.94766761 | 95.08473172 | -1.0492 | 6.26E-06 | - |
| ENSBTAG00000026666 | 583.6202424 | 1462.455097 | -1.3253 | 5.09E-98 | MGC137014 |
| ENSBTAG00000031385 | 32.08658582 | 69.2447257 | -1.1097 | 5.86E-05 | RFWD2 |
| ENSBTAG00000032558 | 23.51938659 | 58.45439352 | -1.3135 | 3.02E-05 | TTC7A |
| ENSBTAG00000033304 | 6.142520208 | 26.69187435 | -2.1195 | 0.00011073 | C27H8orf4 |
| ENSBTAG00000034185 | 312.298659 | 990.4794772 | -1.6652 | 5.23E-91 | EEF1A1 |
| ENSBTAG00000035959 | 40.53255111 | 99.54689917 | -1.2963 | 6.89E-08 | -//- |
| ENSBTAG00000037509 | 17.25563243 | 70.46168046 | -2.0298 | 9.38E-10 | AKR1C4 |
| ENSBTAG00000037570 | 5.697995719 | 70.25885467 | -3.6242 | 4.22E-16 | - |
| ENSBTAG00000037673 | 20.16524726 | 49.77344958 | -1.3035 | 0.00012857 | GSTM4 |
| ENSBTAG00000037738 | 38.10787208 | 86.80943937 | -1.1878 | 2.25E-06 | SLC17A4 |
| ENSBTAG00000038058 | 13.86108179 | 137.353627 | -3.3088 | 4.38E-28 | - |
| ENSBTAG00000039643 | 74.88217069 | 304.1981242 | -2.0223 | 6.92E-37 | - |
| ENSBTAG00000039647 | 242.7911935 | 622.107272 | -1.3574 | 3.86E-44 | MGC152010 |
| ENSBTAG00000039664 | 0 | 22.2297069 | -7.5186 | 1.79E-06 | -//- |
| ENSBTAG00000039719 | 69.66911078 | 159.9889855 | -1.1994 | 1.01E-10 | PHGDH |
| ENSBTAG00000039808 | 12.44668568 | 92.73195252 | -2.8973 | 1.60E-17 | SERPINA6 |
| ENSBTAG00000039928 | 7.67815026 | 151.3891718 | -4.3014 | 3.79E-36 | - |
| ENSBTAG00000039991 | 12.85079886 | 70.25885467 | -2.4508 | 9.60E-12 | - |
| ENSBTAG00000040298 | 48.53399191 | 143.5195311 | -1.5642 | 1.55E-13 | GSTT1 |
| ENSBTAG00000043546 | 305.4287351 | 672.6514596 | -1.139 | 2.42E-37 | MT-ND6 |
| ENSBTAG00000043558 | 1510.777092 | 3964.676339 | -1.3919 | 2.35E-281 | ND1 |
| ENSBTAG00000043563 | 1373.823138 | 2790.558389 | -1.0224 | 1.44E-127 | ND5 |
| ENSBTAG00000043564 | 0 | 87.29622127 | -11.077 | 6.44E-14 | MT-ATP8 |
| ENSBTAG00000043568 | 775.2911197 | 3819.737028 | -2.3007 | 0 | MT-ND3 |
| ENSBTAG00000045728 | 3.434961958 | 84.37552986 | -4.6185 | 1.87E-21 | SCD |
| ENSBTAG00000046155 | 375.623193 | 980.7032739 | -1.3845 | 1.92E-70 | RGN |
| ENSBTAG00000046668 | 35.5619591 | 95.08473172 | -1.4189 | 1.92E-08 | ANG2 |
| ENSBTAG00000047379 | 25.17625059 | 88.59430635 | -1.8152 | 1.56E-10 | - |
| ENSBTAG00000047547 | 0 | 23.64948745 | -8.1928 | 1.83E-06 | - |
| ENSBTAG00000047957 | 0 | 27.34091689 | -5.0098 | 4.23E-08 | - |
| ENSBTAG00000048275 | 56.93954588 | 114.596573 | -1.0091 | 1.46E-06 | - |
| Novel00129 | 0 | 15.73928153 | -8.6054 | 0.00014221 | -//- |
| Novel00968 | 13.9014931 | 65.14764469 | -2.2285 | 4.98E-10 | -//- |
